# Renal kallikrein excretion and epigenetics in human acute kidney injury: Expression, mechanisms and consequences

**DOI:** 10.1186/1471-2369-12-27

**Published:** 2011-06-16

**Authors:** Sun Woo Kang, Pei-an Betty Shih, Roy O Mathew, Manjula Mahata, Nilima Biswas, Fangwen Rao, Liying Yan, Josee Bouchard, Rakesh Malhotra, Ashita Tolwani, Srikrishna Khandrika, Ravindra L Mehta, Daniel T O'Connor

**Affiliations:** 1 Department of Nephrology, Inje University, Busan, South Korea; 2 Departments of Medicine and Pharmacology, and Institute for Genomic Medicine, University of California at San Diego, CA, USA; 3 Veterans Affairs Medical Center, Albany, NY, USA; 4 EpigenDx, Worcester, MA, USA; 5 Service de Néphrologie, Département de médecine, Hôpital du Sacré-Coeur de Montréal, Université de Montréal, Montreal, Quebec, Canada; 6 Division of Nephrology and Hypertension, Department of Medicine, University of California San Diego Medical Center, San Diego, CA, USA; 7 Division of Nephrology, University of Alabama at Birmingham, AL, USA

## Abstract

**Background:**

Renal kallikrein (KLK1) synthesis and urinary excretion are reportedly diminished during AKI (acute kidney injury) in animal models, and provision of kallikrein abrogates renal injury in this setting, but data in human AKI is limited. Therefore we first examined KLK1 renal excretion in human AKI, and then probed potential endocrine and epigenetic mechanisms for its alterations.

**Methods:**

KLK1 enzymatic activity excretion was evaluated in urine from patients with established or incipient AKI, versus healthy/non-hospital as well as ICU controls. Endocrine control of KLK1 excretion was then probed by catecholamine and aldosterone measurements in established AKI versus healthy controls. To examine epigenetic control of KLK1 synthesis, we tested blood and urine DNA for changes in promoter CpG methylation of the *KLK1 *gene, as well as *LINE-1 *elements, by bisulfite sequencing.

**Results:**

Patients with *early/incipient *AKI displayed a modest reduction of KLK1 excretion, but unexpectedly, *established *AKI displayed substantially *elevated *urine KLK1 excretion, ~11-fold higher than healthy controls, and ~3-fold greater than ICU controls. We then probed potential mechanisms of the change. Established AKI patients had lower SBP, higher heart rate, and higher epinephrine excretion than healthy controls, though aldosterone excretion was not different. Promoter *KLK1 *CpG methylation was higher in blood than urine DNA, while *KLK1 *methylation in blood DNA was significantly higher in established AKI than healthy controls, though *KLK1 *methylation in urine tended to be higher in AKI, directionally consistent with earlier/incipient but not later/established changes in KLK1 excretion in AKI. On multivariate ANOVA, AKI displayed coordinate changes in KLK1 excretion and promoter methylation, though directionally opposite to expectation. Control (LINE-1 repetitive element) methylation in blood and urine DNA was similar between AKI and controls.

**Conclusions:**

Unexpectedly, *increased *KLK1 excretion in AKI patients was found; this increase is likely to be due in part to increments in adrenergic tone during BP depression. Epigenetic changes at *KLK1 *may also play a role in early changes of KLK1 expression and thus AKI susceptibility or recovery.

## Background

The incidence of acute kidney injury (AKI) in hospitalized patients is estimated to be 5-10%, and is much higher in the critically ill [1,2]. Despite the potential for recovery of kidney function, acute kidney injury is associated with substantial morbidity and even mortality. AKI, due to ischemia or nephrotoxic agent exposure, may lead to death or sublethal injury of proximal tubular cells, after which surviving cells may repolarize and/or de-differentiate, proliferate, migrate to denuded areas, re-differentiate, and restore nephron structure (including the tubular epithelium) and function [3,4].

The serine protease kallikrein (KLK1; E.C.-3.4.21.35; OMIM 147910), excreted from kidney into urine, catalyzes the cleavage of low molecular weight kininogen to lysyl-bradykinin (kallidin), which exhibits both vasodilator and natriuretic pharmacological properties in the kidney; if these properties occur *in vivo*, the potential of the system for regulating blood pressure is clear [5].

Renal kallikrein levels were markedly reduced in an aminoglycoside-induced AKI animal model [6], and KLK1 gene transfer protected against aminoglycoside-induced nephropathy by diminishing apoptosis and inflammation [7]. In addition, KLK1 infusion during aminoglycoside treatment attenuated drug-induced renal dysfunction, cortical damage, and apoptosis in the rat [8]. Previously, we have reported that urinary KLK1 excretion was diminished in renal allograft recipients with a clinical diagnosis of acute tubular necrosis (ATN) [9]; since urinary KLK1 originates in the kidney, reduced urinary kallikrein levels may reflect impaired renal function. However, this finding has not been pursued in humans.

In mammals, cytosine methylation occurs almost exclusively at CpG dinucleotides, which are enriched at CpG islands, often are located at 5'-/promoter regions of functional genes [10]. Cytosine methylation may result in transcriptional repression either by interfering with transcription factor binding or by inducing a repressive chromatin structure [11]. Apoptotic pathways are targets for such "epigenetic" silencing, and several apoptosis-linked genes that are regulated directly or indirectly by methylation have been described [12].

In this study, we first probed whether KLK1 excretion is altered in human AKI, and if so what mechanisms (endocrine or epigenetic) might be driving the change. Since kidney repair after injury may recapitulate normal morphogenesis, we hypothesized that urinary kallikrein levels would be associated with severity of AKI and with epigenetic changes in the renal kallikrein-1 (*KLK1*) promoter. We considered that changes in kallikrein excretion or the *KLK1 *promoter might predict renal functional recovery and thus serve as biomarkers of recovery from AKI, thereby facilitating timely diagnosis and treatment. We therefore examined patients with AKI for urinary expression of KLK1 enzymatic activity, as well as genomic DNA from blood and urine for CpG methylation pattern at the *KLK1 *gene promoter.

## Methods

### Established AKI cases

Cases were ascertained from a single center prospective non-concurrent observational cohort of inpatients who were identified as having suffered acute kidney injury (AKI) during a hospital admission. The institutional review board of the University of California, San Diego (UCSD) reviewed and approved the study as well as the consent document. Primary providers referred potentially eligible patients to investigators and study coordinators. Patients were eligible for enrollment if they were greater than 18 years of age and met the serum creatinine criteria for acute kidney injury as set out by the Acute Kidney Injury Network (AKIN) [13]: an abrupt rise by ≥0.3 mg/dl within a 48-hour period. Patients were excluded if they received chronic renal replacement therapy (hemodialysis or peritoneal dialysis) within the 6 months prior to admission; had ever been in receipt of a kidney transplant; pregnant or breast feeding; currently incarcerated or otherwise institutionalized (nursing home, rehabilitation); were placed under hospice/comfort care; or did not have reasonable expectation of survival past the present hospitalization. If eligible, the patient or authorized representative was presented the study and consent for participation was obtained. All relevant patient data were derived from the electronic and paper medical records, as well as direct interview of the patient or surrogate. Basic demographic information and co-morbid conditions were recorded on enrollment. Etiology of AKI was determined by chart review of provider diagnosis, and verification by study personnel; diagnosis followed the categories set by the Project to Improve Care of Acute Renal Disease (PICARD) study investigators [1]. Daily assessment included medication review, physical exam (as recorded in medical record or assessed by study personnel when missing), vital signs, intake and output, and labs. Clinical data elements were collected daily and the need for and utilization pattern of renal replacement therapy was also monitored and recorded. Blood and urine samples were collected at time of entry, daily for 7 days maximum, and at hospital discharge. Twenty-four hour urine collections were performed at study entry and hospital discharge for creatinine/urea clearance (in approximation of glomerular filtration rate - GFR) as well as urinary kallikrein activity. One sample of whole blood was collected for genomic DNA preparation (and genetic analysis of the *KLK1 *locus) at the time of study entry. At the time of discharge, follow-up appointments with either the primary care physician or a nephrologist (not all patients were seen by a nephrologist during the hospitalization), extent of renal recovery, and dialysis dependence (if needed during hospitalization) were ascertained. Recovery: pre-defined study endpoints were 12 months of follow-up, start of maintenance hemodialysis or peritoneal dialysis, receipt of a kidney transplant, or death; recovery of renal function was the primary outcome, defined as a return to within 10% of baseline eGFR or lowest eGFR prior to AKI event. Recovery was assessed at 6 months of follow-up.

### Healthy (non-hospital) controls

In addition, we obtained data for a control group of 38 healthy adult subjects. Each healthy control was selected from only one member from each of 38 twin pair sets. Twin pairs were recruited by a population-based twin registry in southern California [14], and by newspaper advertisement. These twins are of European, African-American, and Asian biogeographic ancestry. Ethnicity was established by self-identification. Self-reported zygosity was confirmed by extensive SNP genotyping. There was no clinical evidence for kidney disease or any other cardiovascular disorder in any of the controls. Untimed (spot) urine collections were performed, and one sample of EDTA-anticoagulated whole blood was collected for genetic analysis of the *KLK1 *locus at the time of study entry.

### Replication: Incipient (early) AKI cases in an intensive care unit (ICU), with ICU control subjects

An additional sample of controls was ascertained for replication of findings, in an ICU setting. In brief, the replication sample consisted of n = 44 subjects ("ICU controls") who did *not *develop AKI during a 7-day observation period after hospital ICU admission, as well as n = 11 subjects (ICU cases) who *did *develop AKI, as defined above. Patients were screened at ICU admission for potential study participation at three academic medical centers (University of California San Diego, University of Alabama, and Université de Montréal) between July 2006 and December 2008. Patients were eligible for enrollment if they were age 18 or older and had a life expectancy of at least 48 hours. Controls were excluded if they had AKI according to the AKIN criteria [13], were admitted to the ICU > 48 hours prior to screening, transferred from another ICU, had a serum creatinine > 2 mg/dl ≤ 3 days before ICU admission, were prisoners, received dialysis within the 12 months prior to admission, had a functioning kidney transplant, were on anticoagulants or warfarin within the last 7 days, suffered from decompensated cirrhosis, had CKD stage 5, were anemic (hemoglobin < 9.0 mg/dl or hematocrit < 27%), or were already enrolled in another research project.

The study was approved by the Institutional Review Boards at each institution, and written informed consent was obtained from all participants or their health care surrogates. Following informed consent, data on past medical history were collected once, and clinical, laboratory and process-of-care elements were collected daily. Each institution's local laboratory measured serum creatinine values. AKI was defined as an increase in serum creatinine level of more than 0.3 mg/dL or more than 50% from a reference creatinine within 48 hours (AKIN criteria)[13]. Patients without AKI within the first 4 days had continued blood and urine samples twice daily, for a total study observation period of 7 days.

### Biochemical assays

Urine was assayed for kallikrein by an alkaline amidolytic activity assay as previously described (4), using the chromogenic substrate S-2266: [D] Val-Leu-Arg-paranitroanilide (Kabi Pharmacia; Franklin, OH, USA) [5,15]. The activity of kallikrein per liter of urine (units per liter, U/L) is calculated from the formula: U/L = (9.55·A), where A = absorbance, after a 30-min assay incubation of the paranitroanilide product in a spectrophotometer at 405 nm [5,15]. Inclusion or exclusion of the kallikrein inhibitor aprotinin (Trasylol, Miles Inc., West Haven, CT, USA) indicated that a fraction of human urinary S-2266 amidolytic activity in the absence of aprotinin was non-kallikrein (likely urokinase) [15]; thus, aprotinin (20 kallikrein inhibitory U/mL) was systematically included in the assay blank, to assure specificity for kallikrein measurement. The inter-assay coefficient of variation was 18.1%, and activities from *n *= 20 samples measured on two separate occasions correlated highly (Spearman *R *= 0.92, *P*< 0.01). In *n *= 87 subjects, activity correlated (Spearman *R *= 0.82, *P*< 0.0001) when results were compared for kallikrein excretion normalized to time versus creatinine excretion. Specificity of the S-2266 amidolytic assay for glandular (KLK1, including renal, pancreas, and salivary) kallikrein activity in urine arises from two features: first, the substrate S-2266 is cleaved only by a particular subset of serine proteases, including KLK1; and second, the inclusion of aprotinin in the assay blank, which specifically inhibits serine proteases including KLK1; nonetheless, only an immunoassay specific for lysyl-bradykinin (the kinin product of KLK1) generation could provide absolute assurance of specificity for KLK1.

Clinical chemistries (serum or urine, electrolytes or creatinine) were measured by spectrophotometric autoanalyzer. Urine aldosterone concentration was determined by enzyme immunoassay (Alpco Diagnostics, Salem, NH, USA). Urine catecholamine concentration was determined by commercial ELISA kit (Labor Diagnostika Nord GmBH & Co. KG, Nordhorn, Germany). Urine values were normalized to creatinine concentration in the same sample.

### DNA extraction and bisulfite treatment of CpG sites

A sample of EDTA-anticoagulated whole blood was obtained from participants and stored at -70°C prior to DNA extraction. Timed and spot urine samples were obtained and frozen at -70°C before assay. Blood DNA was prepared from blood leukocytes with a commercial kit (QIAamp^® ^DNA Mini Kit; Qiagen, USA). Urine DNA was prepared from the urine pellet with spun columns (urine DNA isolation kit; Norgen, Canada). Both DNAs were subjected to sodium metabisulfite (Na_2_S_2_O_5_) treatment (Imprint™ DNA Modification Kit; Sigma, USA), and then eluted in 20 μL elution buffer. Bisulfite converts cytosine residues to uracil, but leaves 5-methylcytosine residues unaffected.

### *KLK1 *promoter CpG methylation analysis

**Figure 1:**
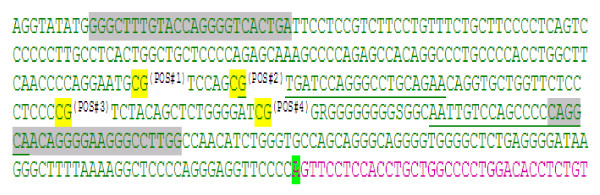
**Map of CpG sites studied in the human *KLK1 *proximal promoter**. Letters highlighted in gray are the target regions complementary to amplified PCR primers. The CpGs analyzed are numbered as Pos#1 - Pos#4 and colored yellow. Green underlined sequences are target regions complementary to sequencing primers. Green highlighted A is the transcriptional initiation ("cap") site. Sequences following the transcription initiation site are colored pink.

Pyrosequencing for allele discrimination (Pyrosequencing; Qiagen, USA) provides real-time extension-based DNA analysis that can evaluate multiple CpG sites [16]. CpG methylation analysis at the 5'/upstream/proximal promoter region of human kallikrein serine protease 1 (*KLK1*) gene was performed. The *KLK1 *gene was analyzed by a single PCR amplicon spanning 4 CpGs in a 263 bp region, with two biotinylated sequencing (extension) primers (Figure 1). The consecutive 4 CpGs were located -203 to -135 bp from the transcription initiation site: sequentially at -203, -196, -154, and -135 bp (Figure 1).

As a control, global CpG methylation analysis was completed using PyroMark LINE-1 reagents (Pyrosequencing; Qiagen, USA). We thus determined the methylation status of three CpG sites in LINE-1 repetitive (LTR-like) elements, wherein methylation levels of CpG sites represent global methylation status across the genome, because of the repetitive nature of LINE-1 elements [17,18].

PCR amplification was performed using 10X PCR buffer, 3.0 mM MgCl_2_, 200 μM of each dNTP, 0.2 μM each of forward and reverse primers, HotStar DNA polymerase (Qiagen, USA) 1.25 U, and ~10 ng of bisulfite-converted DNA per 50 μl reaction. PCR cycling conditions were: 94°C 15 min; 45 cycles of 94°C 30 s, 56°C 30 s, 72°C 30 s; 72°C 5 min; and then products were held at 4°C. The PCR was performed with one of the PCR primers biotinylated to convert the PCR product to single-stranded DNA template. PCR products (each 10 μl) were sequenced by Pyrosequencing PSQ96 HS System (Pyrosequencing, Qiagen, USA). The methylation status of each locus was analyzed individually as a T/C SNP using Pyro-QCpG™ software (Pyrosequencing).

AKI case samples were evaluated at study entry (baseline, time of diagnosis). After bisulfite modification and PCR amplification, *KLK1 *blood DNA promoter methylation data from 13 AKI patients and 30 controls were obtained. *KLK1 *urine DNA promoter methylation data from 9 AKI and 22 controls were available. LINE-1 blood DNA methylation data from 14 AKI patients and 32 controls were available for evaluation. LINE-1 urine DNA methylation data from 15 AKI patients and 32 controls were obtained.

### Statistical analyses

Results are expressed as the mean value ± one standard error of the mean (SEM) for continuous variables. For comparisons of two groups, unpaired two-sided *t*-tests or one-way ANOVA (enabling adjustment for covariates of age, sex, and ethnicity) were performed. Non-parametric Wilcoxon Rank Sum test was used to confirm parametric tests in the face of relatively small sample sizes. Proportions were evaluated by Fisher's exact test (2 × 2 tables) or chi-square test (3 × 2 tables). Statistical analyses were performed in R2.10.1 <http://www.r-project.org/ > or SPSS-17 (Statistical Package for the Social Sciences; Chicago, IL, USA). A *P *value of < 0.05 was considered significant. Multiple linear regression was performed with default criteria of entry (p < 0.05) and exit (p > 0.10) from the multivariate regression model, using stepwise or forward options. Recovery from AKI was pre-defined as return (within 6 months follow-up) to within 10% of baseline eGFR, or lowest eGFR prior to AKI event.

## Results

### Renal KLK1 excretion and eGFR in the 4 subject groups: AKI cases and controls

Demographic and anthropometric description of the study samples is presented in Table 1. Baseline demographic characteristics (age, sex) were similar across groups.

**Table 1:** Clinical characteristics of cases and controls: Established versus incipient AKI cases, and ICU versus healthy controls

		AKI		Controls	
	
	P < 0.05 (*)	Established	Incipient (early, ICU)	ICU	Healthy (non-hospital)
**Characteristics**		n = 20	n = 11	n = 44	n = 38
**Age, years**	*	48.8 ± 3.5	68.1 ± 3.8	52.9 ± 2.3	46.3 ± 1.5
**Sex (male/female)**		15/5	5/6	26/18	28/10
**Ethnicity, n**	*				
White		11	5	28	20
Black		3	4	8	6
Hispanic		5	0	7	6
Other		1	2	1	6
**Lab findings at enrollment**					
sCr, mg/dl	*	2.67 ± 0.43	1.29 ± 0.12	0.81 ± 0.05	0.9 ± 0.04
eGFR, ml/min	*	44.4 ± 7.3	60.9 ± 5.7	98.2 ± 4.9	97.5 ± 3.5
uCr/sCr, ratio	*	42.4 ± 7.2	83.3 ± 18.1	106.6 ± 18.3	119.6 ± 5.2
Urine kallikrein (U/gm creatinine)	*	6.74 ± 1.92	1.17 ± 0.16	2.04 ± 0.47	0.63 ± 0.08
**Vital signs at enrollment**					
Systolic BP, mmHg	0.08	119.8 ± 4.4	126.9 ± 8.7	124.3 ± 3.4	131.4 ± 1.7
Diastolic BP, mmHg	*	70.7 ± 3.4	61.8 ± 5.1	68.6 ± 2.3	74.7 ± 1.5
Heart rate, beats/min	*	89.3 ± 3.6	86.5 ± 7.4	81.3 ± 2.4	68.0 ± 1.6
**Co-morbid conditions (Y/N)**					
Diabetes mellitus		6/14	6/5	12/32	0/38
Hypertension	*	10/10	8/3	20/22	8/30
Coronary artery disease		1/19	4/7	10/34	0/38
Congestive heart failure	*	0/20	3/8	4/40	0/38
Chronic liver disease	*	6/14	0/11	4/40	0/38
Chronic lung disease	*	4/16	3/8	7/37	0/38
Chronic kidney disease	*	8/12	3/8	1/43	0/38
HIV positive		2/18	0/11	3/41	0/38
Malignancy		3/17	1/10	12/32	0/38
Smoker	*	8/12	3/8	16/28	4/34
**Primary treating service, n**					
Medical/Surgical		14/6	11/0	34/10	-
**Other characteristics while hospitalized (Y/N)**					
ICU admission	*	13/7	11/0	44/0	-
Ventilator at enrollment		5/15	3/8	13/31	-
Pressor infusions	*	3/17	9/2	12/32	-
Norepinephrine	*	2/18	8/3	11/33	-
Epinephrine + dopamine	*	1/19	9/2	12/32	-
Diuretics at enrollment	*	3/17	5/6	2/42	-
**AKI outcomes**					
Required dialysis for AKI		3/17	1/10	-	-
Recovery from AKI	*	17/3	11/0	-	-
Remained dialysis- dependent at follow-up		1/19	0/11	-	-
Final eGFR at follow-up		66.5 ± 8.0	76.6 ± 5.5	-	-
eGFR Δ at follow-up		+16.5 ± 2.2	+22.2 ± 5.4	-	-
sCr Δ at follow-up	0.08	-1.0 ± 0.3	-0.25 ± 0.05	-	-

ICU = intensive care unit, AKI = acute kidney injury, n = number of study subjects, BMI = body mass index, s = serum, u = urine, Cr = creatinine, FeNa^+ ^= fractional excretion of sodium, SBP = systolic blood pressure, DBP = diastolic blood pressure, HR = heart rate, bpm = beats per minute. Δ = change at discharge (from initial value). *P values calculated with Fisher's exact test for categorical variables and ANOVA (log-transformed and covariates adjusted: age, sex) for continuous variables. *: Symbol indicates *P *≤ 0.05 comparing across all available groups. Recovery of renal function was the primary outcome, defined as a return to within 10% of baseline eGFR or lowest eGFR prior to AKI event. Plus-minus values are mean ± SEM.

**Figure 2:**
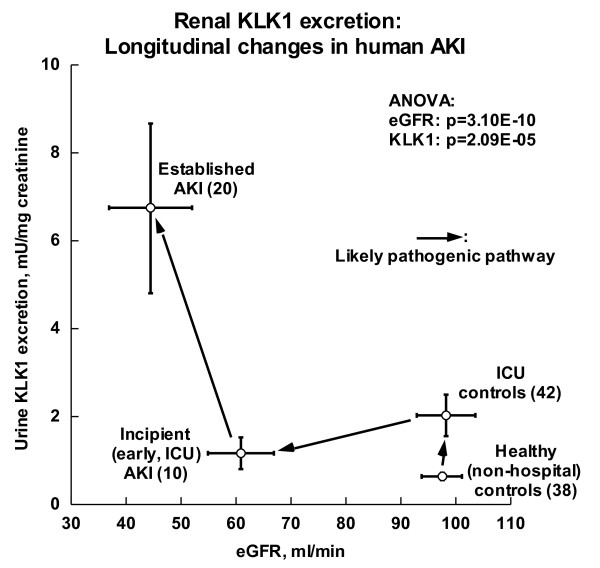
**Urinary kallikrein activity in AKI: Cases versus controls**. Results are shown for both KLK1 excretion and eGFR in 4 groups: established AKI, incipient (early, ICU) AKI, ICU controls, and healthy (non-hospital) controls. Results (shown as mean ± 1 SEM) were analyzed by ANOVA, factoring for age, sex and race.

As compared to healthy/outpatient controls (Table 1, Figure 2), ICU/inpatient controls displayed unaltered eGFR, though a modest ~3.2-fold increase in urinary KLK1 excretion. ICU subjects with incipient AKI had a modest fall in eGFR (down ~38% compared to ICU controls), coupled with a ~43% fall in KLK1 excretion. However, subjects with established (more severe) AKI exhibited a ~6.9-fold increase in KLK1 excretion (p = 2.09E-05), coupled with a further ~27% fall in eGFR (p = 3.10E-10).

### Established AKI: Clinical characteristics

We then turned our attention to clinical features (Table 2) of the AKI subjects that might account for the KLK1 elevation. In clinical laboratory findings at study enrollment (entry), patients with AKI had significantly higher serum creatinine (2.67 ± 0.43 vs. 0.9 ± 0.04 mg/dl; p < 0.001), and fractional excretion of sodium (FeNa^+^; 1.7 ± 0.4 vs. 0.8 ± 0.06%; p = 0.05), than healthy controls, with lower eGFR (44.4 ± 7.5 vs. 97.5 ± 3.6 ml/min; p = 0.0001).

**Table 2:** Mechanistic studies: Characteristics of the AKI study subjects versus healthy controls

Characteristics	Established AKI patients (n = 20)	Healthy controls (n = 38)	P value*
**Age, years**	48.8 ± 3.5	46.3 ± 1.5	0.52
**Sex (male/female), n**	15/5	30/8	1
**Ethnicity, n**			0.6
White	11	20	
Black	3	6	
Hispanic	5	6	
Other	1	6	
**Weight, kg**	91.2 ± 6.2	93.9 ± 3.7	0.293
**BMI, kg/m^2^**	31.1 ± 2.2	27.8 ± 1	0.177
**Laboratory findings at enrollment**			
sCr, mg/dl	2.7 ± 0.47	0.9 ± 0.04	**0.0007**
eGFR, ml/min	44.4 ± 7.5	97.5 ± 3.6	**< 0.0001**
uNa^+^/uCr, mEq/gm	127.6 ± 22.5	125.4 ± 9.4	0.347
uCr/sCr, ratio	42.4 ± 7.2	119.6 ± 11.4	**< 0.0001**
FeNa^+^, %	1.7 ± 0.4	0.8 ± 0.1	**0.05**
**Vital signs at enrollment**			
Systolic BP, mmHg	119.8 ± 4.4	131.4 ± 1.7	**0.02**
Diastolic BP, mmHg	70.7 ± 3.4	74.7 ± 1.5	0.3
Heart rate, beats/min	89.3 ± 3.6	68.0 ± 1.6	**< 0.0001**
**Contributing causes to AKI, n **(with urine KLK1 activity excretion, U/gm creatinine, mean ± SEM)			0.83
Ischemia	7 (6.0 ± 3.7)	-	-
Nephrotoxins	4 (7.2 ± 3.9)	-	-
Septic	1 (4.6)	-	-
Multifactorial causes/other	8 (7.6 ± 2.9)	-	-

AKI = acute kidney injury, n = number of study subjects, BMI = body mass index, s = serum, u = urine, Cr = creatinine, FeNa^+ ^= fractional excretion of sodium, SBP = systolic blood pressure, DBP = diastolic blood pressure, HR = heart rate, bpm = beats per minute, ICU = intensive care unit. *P values were calculated with Fisher's exact test for categorical variables, and ANOVA (log-transformed, adjusted for covariates: age, sex) for continuous variables. Plus-minus values are mean ± one SEM. **Bold**: p ≤ 0.05.

At study enrollment, AKI patients had lower SBP (119.8 ± 4.4 vs. 131.4 ± 1.7 mmHg; p = 0.02) and higher heart rate (89.3 ± 3.6 vs. 68.0 ± 1.6 beats/min; p = 1.73E-05) than healthy controls. Within the AKI group (n = 20), acute kidney injury was attributed to ischemia in 7 patients, nephrotoxins in 4, sepsis in 1, and multifactorial causes in 8 (Table 2); KLK1 excretion did not vary by AKI causal group (ANOVA p = 0.83). Six patients had diabetes mellitus, 10 had hypertension, 1 had coronary artery disease, 6 had chronic liver disease, 4 had chronic lung disease, 8 had chronic kidney disease (previous eGFR < 60 ml/min), 13 required admission to an ICU during hospitalization, 5 required mechanical ventilation at enrollment, and 5 had oliguria at enrollment. At study entry, 3 patients required infusion of vasopressors (2 norepinephrine, 1 dopamine and epinephrine combination), and 3 took diuretics (2 furosemide, 1 thiazide). In the evaluation of primary outcome, 17 patients attained recovery of renal function (see Methods) at 6 months of follow-up (Table 1).

### Established AKI: Kallikrein, catecholamines, and aldosterone

Here we probed potential hormonal mechanisms whereby KLK1 excretion was elevated in established AKI, focusing on such known KLK1 stimulators as catecholamines [19] and aldosterone.

### Urinary kallikrein enzymatic activity

From the 20 established AKI patients and 38 healthy controls, urine was available for kallikrein measurement (Table 3) in 18 patients and 37 controls. Unexpectedly, established AKI subjects displayed substantially *elevated *kallikrein excretion (Figure 2, Table 1), about ~10 times higher than that of controls (activity: 6132.9 ± 2302 vs. 623.0 ± 88.2 mU/L, p < 0.001; urine kallikrein activity/creatinine ratio: 6.74 ± 1.92 vs. 0.63 ± 0.08 U/gm, p < 0.001). To exclude the possibility that diuretic treatment at study entry increased urinary kallikrein excretion [20], we conducted statistical analysis again by exclusion of the 3 diuretic cases (Tables 1); urinary kallikrein excretion remained significantly different between AKI and controls (urine kallikrein/urine creatinine ratio: 7.14 ± 2.18 vs. 0.63 ± 0.08 mU/mg; p = 0.001). We measured the urinary non-kallikrein amidolytic activity (likely urokinase) by inclusion or exclusion of the kallikrein inhibitor aprotinin. The percentage of kallikrein activity within total S-2266 amidolytic activity was not different between AKI patients and controls (78.0 ± 4.8% vs. 69.0 ± 2.4%; p = 0.072).

**Table 3:** Mechanistic studies: Urinary biochemistries in established AKI cases versus healthy controls

	AKI patients	*n available*	Healthy controls	*n available*	T-test P	ANOVA P†	Wilcoxon rank P
**Urine kallikrein (U/gm creatinine)**	6.74 ± 1.92	18	0.63 ± 0.08	37	**0.0058**	**0.00029**	**0.0012**
**Urine aldosterone (pg/mg creatinine)**	9269.9 ± 2652.31	16	12982.4 ± 2619.78	38	0.325	0.65	0.1282
**Urine epinephrine (ng/mg creatinine)**	20.1 ± 2.4	14	7.48 ± 1.07	32	**1.11E-06**	**0.00016**	**1.79E-05**
**Urine norepinephrine, (ng/mg creatinine)**	37.15 ± 8.14	14	26.85 ± 3.2	32	0.344	0.16	0.4424
**Urine kallikrein/urine aldosterone ratio (mU/μg)**	872.0 ± 277.5	15	160.5 ± 76.7	38	**0.00027**	**8.45E-05**	**3.62E-05**
**Urine kallikrein/urine epinephrine ratio (mU/ng)**	0.32 ± 0.13	13	0.18 ± 0.04	31	0.8465	0.8	0.899

Plus-minus values are covariate-adjusted mean ± one SEM. N is the number of study subjects available to conduct each experiment. †Results are analyzed by one-way ANOVA, factoring for age, sex and race. **Bold**: p ≤ 0.05.

Since black and white subjects differ in reported KLK1 excretion [5,15], we evaluated the role of ethnicity in our samples (Table 1). Although cases and controls each included several biogeographic ancestries, KLK1 excretion did not differ significantly in black versus white cases (p = 0.26) or black versus white controls (p = 0.69), perhaps reflecting the relatively small sample sizes. Disease analyses were adjusted for biogeographic ancestry as a covariate.

### Urinary aldosterone and catecholamine excretions

**Figure 3:**
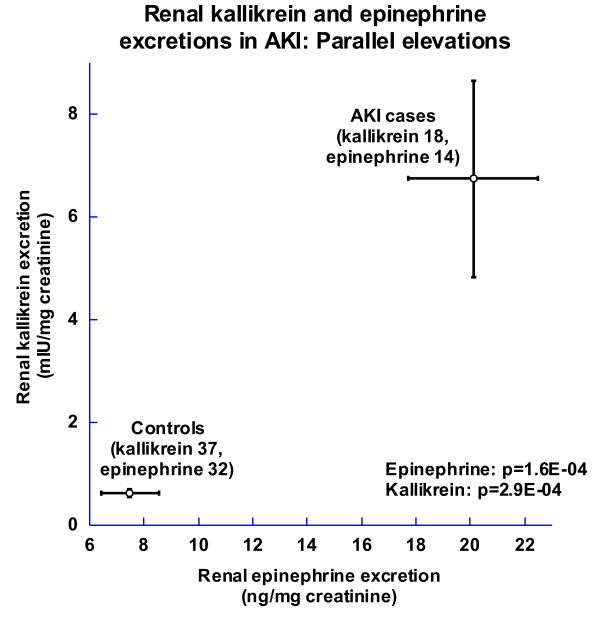
**Coordinate effects of AKI on kallikrein and epinephrine excretion**. Results (shown as mean ± 1 SEM) were analyzed by one-way ANOVA, factoring for age, sex and race. The numbers studied for kallikrein are 18 established AKI patients and 37 healthy controls. The numbers studied for epinephrine are 14 established AKI patients and 32 healthy controls.

We measured urinary aldosterone, epinephrine and norepinephrine excretions (Table 3), since these hormones are known to increase urinary kallikrein. Established AKI subjects exhibited substantially elevated epinephrine excretion, ~2.7 times higher than that of healthy controls (Table 3, Figure 3; 20.1 ± 2.4 vs. 7.48 ± 1.07 ng/mg creatinine; ANOVA p = 0.00016). Urinary kallikrein/epinephrine ratio did not differ between groups (Table 3); parallel elevations of KLK1 and epinephrine suggest that the epinephrine excess in AKI may be sufficient to account for the increased KLK1. To exclude the possibility that epinephrine infusion (in one case, Tables 1) increased urinary excretion, in a subsequent analysis we excluded that case, but urinary epinephrine remained different between AKI and controls (19.99 ± 2.62 vs. 7.48 ± 1.07 ng/mg creatinine; ANOVA p = 0.001). Indeed, Figure 3 illustrates parallel elevations of renal kallikrein and epinephrine excretion in the AKI group.

Urinary aldosterone excretion did not differ between established AKI and healthy controls (Table 3) (9269.9 ± 2652.31 vs. 12982.4 ± 2619.78 pg/mg creatinine; ANOVA p = 0.65). Since urinary kallikrein/aldosterone ratio was also elevated in AKI (Table 3), increments in aldosterone could not explain the rise in KLK1 excretion in AKI. Urinary norepinephrine excretion did not differ in AKI (Table 3) (37.15 ± 8.14 vs. 26.85 ± 3.20 ng/mg creatinine; ANOVA p = 0.16); nor did urine kallikrein/norepinephrine excretion ratios.

### Urine albumin excretion

Quantitative urine albumin excretion was evaluated in established AKI cases and healthy controls. Albumin values in cases ranged from 2.0-4490 mg/gm creatinine (mean, 1090 mg/gm), but kallikrein and albumin excretions did not correlate (Pearson r = 0.006, p = 0.54), rendering it unlikely that elevated kallikrein activity in AKI arose simply from pathological excretion of plasma proteases. In healthy controls, albumin excretion was 6.27 ± 0.39 mg/gm creatinine.

### ICU controls: Urinary kallikrein activity and clinical findings

44 "ICU controls" were available to evaluate the specificity of urinary kallikrein elevation in AKI. Table 1 shows demographic, laboratory and clinical findings of these ICU controls. The kallikrein increment in AKI persisted when studied with ICU controls (6.74 ± 1.92 vs. 2.04 ± 0.47 U/gm creatinine; p = 0.028), though kallikrein excretion was modestly elevated in ICU- compared to healthy controls (2.04 ± 0.47 vs. 0.63 ± 0.08 U/gm creatinine; p = 0.005).

Compared with established AKI, ICU controls had significantly lower serum creatinine (0.81 ± 0.05 vs. 2.67 ± 0.43 mg/dl; p = 0.0005). Compared with healthy controls, ICU controls were older (54.2 ± 2.2 vs. 46.3 ± 1.5 years; p = 0.005), with higher heart rate (81.3 ± 2.4 vs. 68.0 ± 1.6 bpm; p < 0.0001), but lower DBP (68.6 ± 2.3 vs. 74.7 ± 1.5 mmHg; p = 0.029). Even though not significantly different, SBPs of ICU controls tended to be lower than those in healthy controls (124.3 ± 3.4 vs. 131.4 ± 1.7 mmHg; p = 0.066). The ICU controls had variable primary diseases (Table 1). 13 among them required mechanical ventilation, while 12 required vasopressor infusion during ICU admission (Table 1).

### *KLK1 *promoter DNA CpG methylation patterns

**Figure 4:**
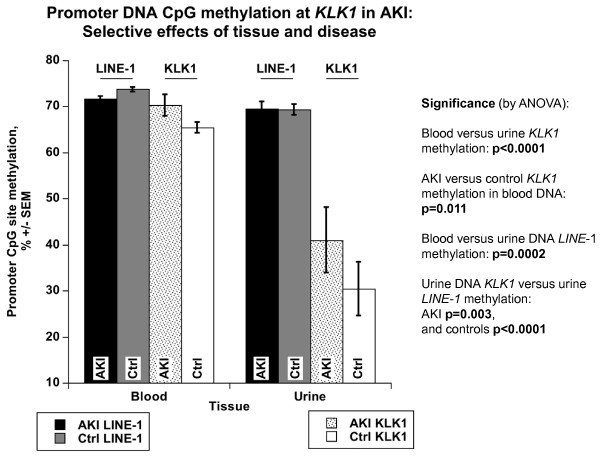
**CpG methylation analyzed by bisulfite sequencing: Results at the *KLK1 *promoter, as well as a global control (LINE-1 repetitive elements), in genomic DNA from urine or blood (mononuclear cells)**. Results (shown as mean ± one SEM), from established AKI cases or healthy controls, were analyzed by ANOVA, factoring for age, sex and race. LINE-1 reagents (Pyromark, Biotage) were used to analyze the 3 CpG sites in LINE-1 repetitive elements, while the *KLK1 *gene was analyzed by a separate PCR covering 4 promoter CpGs. Promoter *KLK1 *specific methylation was substantially higher in blood than urine DNA (blood 66.38 ± 1.00 vs. urine 33.43 ± 4.67%; ANOVA p < 0.0001). Promoter *KLK1 *methylation in blood DNA was higher in AKI than controls (AKI, 70.32 ± 2.27 vs. controls, 65.36 ± 1.05%; ANOVA p = 0.011). Promoter *KLK1 *methylation in urine DNA did not differ in AKI versus controls (AKI, 40.95 ± 7.06 vs. controls, 30.35 ± 5.88%; ANOVA p = 0.22). Global LINE-1 methylation was greater in both blood than urine DNA (blood 73.11 ± 0.38 vs. urine 69.37 ± 0.86%, p = 0.0002). LINE-1 methylation in blood DNA did not differ in AKI and controls (AKI, 71.71 ± 0.44 vs. controls, 73.67 ± 0.41%; ANOVA p = 0.08). LINE-1 methylation in urine DNA did not differ in cases/controls (AKI, 69.53 ± 1.54 vs. controls, 69.29 ± 1.05%; ANOVA p = 0.79).

*KLK1 *promoter CpG methylation (positions in Figure 1) was studied in established AKI and healthy controls. Promoter *KLK1 *CpG methylation (Figure 4) was higher in blood than urine DNA (blood 66.38 ± 1.00 vs. urine 33.43 ± 4.67%; ANOVA p < 0.0001). Promoter *KLK1 *methylation in blood DNA was also higher in AKI than controls (70.32 ± 2.27 vs. 65.36 ± 1.05%; ANOVA p = 0.011), while promoter *KLK1 *methylation in urine DNA trended to be higher in AKI than controls (40.95 ± 7.06 vs. 30.35 ± 5.88%; ANOVA p = 0.22; Figure 4).

Global CpG methylation, examined by LINE-1, was high in both blood and urine, though even higher in blood (blood 73.11 ± 0.38 vs. urine 69.37 ± 0.86%, p = 0.0002). LINE-1 CpG methylation in blood DNA was similar between AKI and controls (71.71 ± 0.44 vs. 73.67 ± 0.41%; ANOVA p = 0.08), as was LINE-1 methylation in urine DNA (AKI 69.53 ± 1.54 vs. control 69.29 ± 1.05%; ANOVA p = 0.79) (Figure 4). In AKI blood DNA, *KLK1*-specific methylation was similar to global LINE-1 methylation (70.32 ± 2.27 vs. 71.71 ± 0.44%; p = 0.56). In controls, however, *KLK1 *CpG methylation in blood DNA was significantly lower than global LINE-1 methylation in blood DNA (65.36 ± 1.05 vs. 73.67 ± 0.41%; p < 0.0001) (Figure 4). In both AKI and controls, *KLK1 *specific methylation in urine DNA was significantly lower than global LINE-1 methylation in urine DNA (AKI 40.95 ± 7.06 vs. 69.53 ± 1.54%; p = 0.003; controls 30.35 ± 5.88 vs. 69.29 ± 1.05%; p < 0.0001) (Figure 4).

**Figure 5:**
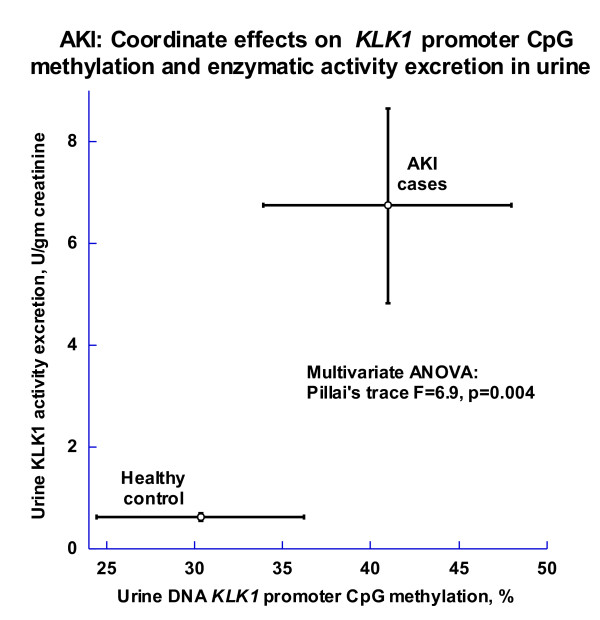
**AKI: Coordinate effects of disease on *KLK1 *promoter CpG methylation and KLK1 enzymatic activity excretion in urine**. The multivariate analysis compared established AKI cases with healthy controls.

Urine *KLK1 *promoter CpG methylation did not predict KLK1 activity excretion. However, in a multivariate analysis, disease status jointly predicted both *KLK1 *promoter methylation and enzyme activity excretion, with higher values for each in cases (multivariate p = 0.004; Figure 5. Since increased KLK1 promoter methylation would be predicted to decrease gene expression, this joint effect cannot explain (and indeed runs counter to) the elevated KLK1 excretion observed in our AKI cases (Figure 5).

## Discussion

### Overview

Several previous lines of investigation link alterations in KLK1 expression to AKI, in both experimental animals and humans. A decrease in urinary kallikrein excretion occurs in rodents with AKI after methemoglobin [21]; rats treated with aminoglycosides have dramatically reduced levels of urinary kallikrein [6], and a transient decrease in urinary kallikrein excretion occurs during chromate-induced AKI in the rat [22]. Renal kallikrein mRNA expression was specifically reduced in the post-ischemic rodent kidney, with persistently altered expression even after functional recovery from ischemic acute renal failure [23]. In humans, we reported previously that urinary kallikrein excretion was diminished in acute tubular necrosis (ATN) after renal transplantation [9].

Studies in rodents suggest beneficial effects of exogenous KLK1 replacement in the setting of experimental AKI. *KLK1 *gene transfer protected against aminoglycoside-induced nephropathy, with inhibition of apoptosis and inflammation [7]. *KLK1 *replacement after gentamicin attenuated drug-induced renal dysfunction, cortical damage, and apoptosis in the rat [8]. Furthermore, KLK1 reduced gentamicin-induced renal dysfunction and fibrosis, with decreased myofibroblast and collagen accumulation [8]. These findings indicate that the renal kallikrein/kinin system prevents and promotes recovery of aminoglycoside-induced renal injury by inhibiting apoptosis, inflammatory cell recruitment, and fibrotic lesions.

Thus, we expected that AKI patients would have *diminished *urinary kallikrein excretion, since urinary kallikrein originates in the kidney; further, we anticipated that kallikrein increments might be associated with superior outcomes in AKI. Unexpectedly our *established *AKI subjects displayed substantially *elevated *(by as much as ~11-fold; ANOVA p = 0.00029; Figure 2; Tables 1,3) kallikrein excretion.

### Origin of increased KLK1 excretion in established AKI

Although previous reports of KLK1 excretion in AKI indicated *diminished *excretion in both rodent models [6,21,22] and AKI/ATN in the setting of human renal transplantation [9], we noted a ~11-fold *elevation *of KLK1 excretion in our established AKI subjects (Tables 1-3; Figure 2). Why might KLK1 excretion be elevated in human AKI? Here we examined hormonal factors known to increase KLK1 excretion: catecholamines and aldosterone [5,19,20,24-26]. We found that an elevation in epinephrine excretion paralleled that for kallikrein (Figure 3), while norepinephrine and aldosterone excretions were unchanged (Table 3). In experimental animals, kallikrein excretion is regulated by adrenergic receptors, with stimulation by β-receptors and inhibition by α-receptors [19]. While diuretics can also elevate kallikrein excretion [20,27], the KLK1 excretion increment in AKI persisted after exclusion of the 3 subjects on diuretics.

**Figure 6:**
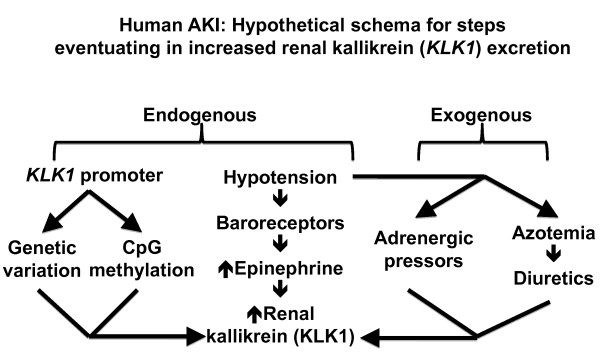
**Hypothetical schema integrating experimental findings in this study of KLK1 in AKI**. This diagram is presented not as established fact, but rather to generate hypotheses for further investigation. *Endogenous *factors may influence KLK1 synthesis and renal excretion: lower BP in AKI may activate baroreceptors, thus increasing endogenous secretion of epinephrine, thereby increasing both heart rate and urinary kallikrein excretion. *KLK1 *promoter genetic variants or CpG methylation can influence renal kallikrein production. Finally, *exogenous *factors such as adrenergic pressor infusions or diuretic treatment can also increase renal kallikrein production; indeed, since a subset of our AKI cases received such treatments (Table 1), we cannot exclude this possibility.

Why was epinephrine elevated in our AKI subjects? AKI patients had lower systolic BP than controls (119.8 ± 4.4 vs. 131.4 ± 1.7 mmHg; p = 0.02) and higher heart rate (89.3 ± 3.6 vs. 68.0 ± 1.6 bpm; p = 1.73E-05) (Table 2). While the mechanism cannot be readily probed in the setting of acute human illness, we suspect that that lower BP in AKI may stimulate baroreceptors, with resulting increase in endogenous production of epinephrine, and consequently increased heart rate and kallikrein excretion (Figure 6).

### *KLK1 *epigenetics

Promoter *KLK1 *specific CpG methylation was higher in blood than urine DNA (blood 66.38 ± 1.00 vs. urine 33.43 ± 4.67%; ANOVA p < 0.0001; Figure 4), consistent with kidney-specific expression of the *KLK1 *gene, in that renal kallikrein is synthesized in the distal tubule and released into urine and peritubular interstitium [20], and cytosine methylation results in transcriptional repression either by interfering with transcription factor binding or by inducing a repressive chromatin structure [11].

*KLK1 *promoter methylation in blood DNA was higher in AKI than controls (70.32 ± 2.27 vs. 65.36 ± 1.05%; p = 0.011), and there was also a trend towards higher urine *KLK1 *methylation in AKI than controls, but the difference was not significant (40.95 ± 7.06 vs. 30.35 ± 5.88%; p = 0.22). While a multivariate analysis indicated a joint effect of AKI on both KLK1 excretion and urine *KLK1 *CpG methylation (Figure 5), elevated *KLK1 *methylation would be predicted to *decrease *KLK1 excretion in AKI, as occurs in *early/incipient *AKI (Figure 2). Increased KLK1 excretion in *later/established *AKI (Figure 2) thus highlights the influence of epinephrine (Figure 3) to elevate KLK1, even in the face of opposing epigenetic influence.

LINE-1 methylation results enabled comparisons of *KLK1 *to global genomic patterns of CpG methylation [18]. In AKI blood DNA, *KLK1*-specific methylation was similar to global LINE-1 methylation (70.32 ± 2.27 vs. 71.71 ± 0.44%; p = 0.56). In control blood DNA, however, *KLK1*-specific methylation was lower than global LINE-1 methylation (65.36 ± 1.05 vs. 73.67 ± 0.41%; p < 0.0001) (Figure 4).

We investigated the 4 most proximal consecutive CpG sites in *KLK1 *promoter (Figure 1). This proximal promoter region is unusually polymorphic, containing a poly-guanine length polymorphism coupled with multiple base-substitution variants that constitute at least ten different alleles or haplotypes [28]. Functional/transfection analysis of several alleles in this region suggests that different variants lead to alterations in expression of the *KLK1 *gene [29]. Since genetic variation may contribute to AKI susceptibility [30], this hypothesis warrants future studies of *KLK1 *promoter variants in larger cohorts, assessing the effects of such variants on both susceptibility and recovery in AKI, since *exogenous *KLK1 does exert protective effects against aminoglycoside-induced AKI [7,8].

It should be noted that the sources of DNA for these epigenetic studies in blood and urine are likely to be heterogeneous - blood DNA could emerge from any leukocyte subpopulation, while DNA in urine can emerge from cell types other than renal. Nor have we established whether the promoter CpG methylation events we observed have functional consequences for transcription.

### Advantages and limitations of this study

Our conclusions are derived from analysis of four subject groups (Figure 2): two degrees of AKI (established versus incipient/early), and two kinds of controls (ICU versus healthy/non-hospital). Furthermore, we were able to probe clinical and biochemical characteristics of cases and controls to identify elevated epinephrine as a likely driver of increased KLK1 excretion (Figure 3, Table 3). While we were able to evaluate epigenetic factors in control of KLK1 excretion in the form of promoter CpG methylation (Figures 1, 4), we did not study other epigenetic mechanisms, such as histone modifications, nor could we probe gene expression more directly by evaluating transcript abundance in tissue, since biopsies would have been hazardous. Furthermore, the established AKI cases were ascertained at a later time point than either AKI cases or controls in the ICU cohorts. Finally, the results would benefit from replication, given the numbers of subjects studied (Figure 2, Table 1), as well as evaluation of additional mediators of AKI risk and repair.

## Conclusions

In conclusion, human patients with established AKI display an unexpected *increase *in urinary KLK1 enzymatic activity excretion (Figure 2); the effect is reproducible across control groups, and seems to be driven by epinephrine excess in the setting of hemodynamic instability (Figures 3, 6). AKI and controls differed in *KLK1 *promoter CpG methylation in blood DNA (AKI > controls), and *KLK1 *CpG methylation differed systematically from global control (LINE-1 element) methylation, suggesting a potential role of epigenetic factors in AKI susceptibility (Figure 4).

## List of Abbreviations

AKI: Acute Kidney Injury; ATN: Acute Tubular Necrosis; BP: Blood Pressure; CpG: 5'-Cytosine-phosphate-Guanine-3'; KLK1: Kallikrein-1 (glandular/renal kallikrein); LINE-1: Long Interspersed Nuclear Element, type-1.

## Competing interests

The authors declare that they have no competing interests.

## Authors' contributions

SWK carried out the molecular genetic studies, analyzed all data and drafted the manuscript. PBS performed the statistical analysis. ROM was in charge of the patient database and the design of the study. MM, SK and NB carried out the biochemical assays. FR was in charge of the healthy control database. LY performed the methylation analysis. JB, RM, and AT were in charge of the ICU patient database. RLM and DTOC were involved in the design of the study, analyzed all data and responsible for the project. All authors read and approved the final manuscript.

## Pre-publication history

The pre-publication history for this paper can be accessed here:

http://www.biomedcentral.com/1471-2369/12/27/prepub
